# Evaluation of an Aqueous-Ethanolic Extract from *Rosmarinus officinalis* (Rosemary) for its Activity on the Hormonal and Cellular Function of Testes in Adult Male Rat

**Published:** 2013

**Authors:** Hamed Heidari-Vala, Reza Ebrahimi Hariry, Mohammad Reza Sadeghi, Mohammad Mehdi Akhondi, Marefat Ghaffari Novin, Mahnaz Heidari

**Affiliations:** a *Reproductive Biotechnology Research Center, Avicenna Research Institute, ACECR, Tehran, Iran.*; b *Department of Pharmacology, Faculty of Medicine, Gazi University, Ankara, Turkey.*; c *Monoclonal Antibody Research Center, Avicenna Research Institute, ACECR, Tehran, Iran. *; d *Infertility and Reproductive Health Research Center, Shahid Beheshti Medical University, Tehran, Iran.*; e *Nanobiotechnology Research Center, Avicenna Research Institute, ACECR, Tehran, Iran.*

**Keywords:** Sperm, Testosterone, *Rosmarinus officinalis*, Rat, Extract

## Abstract

*Rosmarinus officinalis *has been used in traditional medicine extensively. This study evaluated the hormonal and cellular effects of *Rosmarinus officinalis *extract on testes of adult rats. Thirty male Wistar rats (in three groups) received 50 or 100 mg/Kg b.w of *Rosmarinus officinalis *extract (made from the plant’s leaves, flower and stem) (treatment groups) and 10 mL/Kg b.w normal saline (control group) respectively, on a daily bases by gavage route for 60 days. Then, spermatological properties, histometric parameters and sperm dynamics, testis and body weight, testicular cell population and serum testosterone level were analyzed by an acceptable method. Results showed that the mean serum testosterone level was decreased significantly in both treatment groups (50 and 100 mg/Kg b.w) during the experiment time, compared with control group (p < 0.05). However, *Rosmarinus officinalis *did not change the total count, motility and viability of sperm. In addition, *Rosmarinus officinalis *at both doses did not change body and testes weight and their ratio. Furthermore, *Rosmarinus officinalis *increased the number of Spermatogonia at both doses, Spermatocyte at doses of 50 mg/Kg b.w, Leydig cell and Spermatid at dose of 100 mg/Kg b.w significantly (p < 0.05). *Rosmarinus officinalis *did not significantly affect the number of Spermatozoid and Sertoli cells. In conclusion, it seems that* Rosmarinus officinalis *may have some hormonal and cellular effects on the testes which can contribute the spermatogenesis process in rat. *Rosmarinus officinalis *may have antiandrogenic effect potentially indicating the possibility of developing herbal male contraceptive.

## Introduction

Nowadays, finding the orally safe and effective contraceptive agents is an important necessity. On the other hand, there are some ethnomedicinal plants which are used in traditional medicine

without any information about their side effects on reproductive functions. *Rosmarinus officinalis* (Rosemary) which belongs to the Lamiaceae family is an aromatic plant. Anthropologists and archaeologists have found evidence that *Rosmarinus officinalis *herbs have been used as medicinal, culinary and cosmetics in ancient Egypt, Mesopotamia, China and India (1). The plant is an evergreen, branched subshrub, 50 to 150 cm high with erect, climbing or occasionally decumbent brown branches. The leaves are linear, coriaceous, entire-margined, tomentose, light green and somewhat rugose above. The medicinal parts are the oil extracted from the leaves and the leafy stems, the flowering, dried twig tips, the dried leaves, the fresh leaves, the fresh aerial parts collected during flowering and the flowering branches. The plant is indigenous to the Mediterranean region and Portugal and is cultivated there as well as on the Crimea, in the Transcaucasus region, Central Asia, India, Southeast Asia, South Africa, Australia and the USA (2). Active components of extracts made from the leaves include caffeic acid derivatives, diterpenes (bitter), flavonoids, triterpenes and volatile oil (3-4). The plant is mildly antimicrobial (5) and antiviral (6). *Rosmarinus officinalis* is used internally for dyspeptic disorders and externally for hypotonic circulatory disorders and rheumatic conditions. It is also used in traditional medicine for digestive symptoms, headaches and migraine, dysmenorrhea, amenorrhea and oligomenorrhea, states of exhaustion, dizziness and poor memory. It is used externally as a poultice for poorly healing wounds, for eczema, as an analgesic for injuries of the mouth and throat, topically for myalgias, intercostal neuralgia and sciatica. However, it should not be used during pregnancy. No health hazards or side effects are known in conjunction with the proper administration of designated therapeutic dosages. Contact allergies have been observed on occasions. Very large quantities of rosemary leaves misused for the purpose of abortion, can lead to deep coma, spasm, vomiting, gastroenteritis, uterine bleeding, kidney irritation, and death in humans (2). The aim of this study was to evaluate the effects of *Rosmarinus officinalis *extract on the hormonal and cellular function of testes in adult rats.

## Experimental

*Plant materials*

The whole plant of *Rosmarinus officinalis *was collected freshly from a cultivation farm of the north of Iran in the year of 2009 (May July). The plant was identified and authenticated by Dr. Abrahmi (the senior botanist of the Department of Botany, University of Tehran) and a voucher specimen was deposited accordingly in the herbarium of the same department. The medicinal parts of plant, the fresh leaves and the whole plant (2), were separated, dried in shade, pulverized by a mechanical grinder and passed through 40-mesh sieve and stored in airtight container for further use.

*Preparation of plant extract*

About 500 g powdered dry parts of the plant were extracted successively with 70% v/v ethanol at 68°C in Soxhlet apparatus. The extracts were collected in 5 L individual conical flasks, filtered, and the solvent was evaporated to dryness under reduced pressure in an Eyela Rotary Evaporator

(Japan) at 40-45°C and were stored in a vacuum desiccator. The dark green liquid extract so obtained was concentrated under vacuum and the resulting dried extract was lyophilized and preserved in a refrigerator at 4°C until use for the experiments. The extract was dissolved in 1% normal saline and was used for experimental purpose.

*Animals*

Adult Wistar strain male rats (25-30 g, 8-9 week) were obtained from the Pastor Institute (Iran). The animals were housed in colony rooms with 12/12 h light/dark cycle at 23 ± 2°C and had free access to water and food (standard pellet feed) according to the manufacturer’s instructions. All

experimental procedures described were followed the CPCSEA rules. All animals were transferred to an individual small cage 45 min before the examination and also were used only one time.

*Design of experiment*

Thirty healthy male Wistar adult rats were selected and divided into three groups containing ten rats each and treated as follows: Groups A and B received *Rosmarinus officinalis *at the dose of 50 and 100 mg/Kg b.w respectively and group C received normal saline (10 mL/Kg b.w) as control group. All doses were administered orally by gavage route on consecutive days for 60 days and all treatment and control groups received their own normal diet. Blood samplings were recorded, prior to, in the middle of (30^th^ day) and after the treatment (60^th^ day). Blood was obtained by tail vein from these animals for the analysis of hormonal profile of testosterone. Blood samples were centrifuged to separate the serums of samples. The serums were kept at - 20°C. At the end of the period, testosterone hormone of all samples was measured by chemiluminescence method. At the end of the experimental period, both control and experimental groups were given anesthesia under mild sodium pentobarbital 24 h after the last dose and 18 h after fasting. Rats were euthanatized for the following investigations.

*Gravimetric analysis of body and testes*

The testes were dissected out, trimmed off from adherent fats and weighed and recorded. Gained weight was divided into whole body weight and obtained result was multiplied to 100 and the statistical analysis was done.

*Spermatological studies*

Vasa deferentia were dissected out, divided to small equal pieces by a scissor, released in 5 mL of normal saline (37°C) and shacked slowly for 5 min to free and float the sperm. A drop was picked from this suspension to investigate the percentage of motile sperm using an optical microscope at 40X. Eosine-Nigrosin dye (Sigma, England) was used to determine the live sperm.

Cell membrane of dead sperm absorbs the dye while cell membrane of live sperm prevents dye entrance into the cell. Morphologically, abnormal sperms were counted. After recording the physical characteristics’ appearance, volume and motility, samples were evaluated for spermatozoa concentration. Improved Neubauer hemocytometer was followed for spermatozoa concentration measurement. All samples were analyzed by one person to avoid person to person variation. A drop of the sample was placed on the Neubauer slide. The grid was located with 200X magnification under a phase contrast microscope. Number of spermatozoa was counted in 16 squares with the help of manual counter. Obtained numbers were multiplied to 5000. Concentration of spermatozoa was calculated as described (7). Data were statistically analyzed using single factor analysis of variance. All spermatological parameters were repeated in order to ascertain the nature of action of extract, *i.e. *reversible or irreversible.

*Statistical analysis*

Results are expressed as mean ± SEM and all statistical analyses were performed using SPSS 13 (Statistical Package for Social Science) and Paired t-test was used to determine the association of each factor and p < 0.05 was considered statistically significant.

## Results and Discussion

*Effect on testosterone levels*

The measurement of testosterone levels of serum from rats showed that there was not a significant difference at both doses of *Rosmarinus* *officinalis *in comparison with control group at the early period of experiment. Nevertheless, at the mid-term of experiment, testosterone level was decreased significantly at dose of 100 mg/Kg b.w (3.2300 ± 0.1959 pg/mL), when compared with control group. This decreasing was not significant at dose of 50 mg/Kg b.w. Serum testosterone levels were decreased significantly at both doses of 50 and 100 mg/Kg b.w at the late period of experiment (3.1514 ± 0.4060 pg/mL and 2.4457 ± 0.2219 pg/mL, respectively) in comparison with control group (Table 1). 

**Table 1:** Effect of *Rosmarinus officinalis *on mean serum testosterone level (pg/mL) in rats prior to, in the middle of (30^th^ day) and after the treatment (60^th^ day

**Treatment**	**Dose**	**Early period**	**Mid-term**	**Late period**
Control group	10 mL/Kg b.w	4.1575 ± 0.3120	4.1575 ± 0.3214	4.4113 ± 0.3344
Group A (Rosmarinus officinalis)	50 mg /Kg b.w	4.1471 ± 0.2468	3.7114 ± 0.3492	3.1514 ± 0.4060*
Group B (Rosmarinus officinalis)	100 mg/Kg b.w	4.0757 ± 0.0763	3.2300 ± 0.1959*	2.4457 ± 0.2219*

*Rosmarinus officinalis *extracts were administered orally by gavage route for 60 days. Values are the mean ± SEM. Ten rats were included per group. *p < 0.05; ** p < 0.01; *** p < 0.001, significantly compared to control group, Paired t-test

*Sperm properties*

*Effect on epididymal sperm count*

Results showed that the Vasa deferentia sperm count was not significantly changed when treated with *Rosmarinus officinalis *at both dose levels. That is, *Rosmarinus officinalis *at the doses of 50 and 100 mg/Kg b.w showed about 58.30 ± 0.33 × 10^6^ and 60.10 ± 0.19 × 10^6^ sperm/ mL, respectively, in comparison with control (59.50 ± 0.29 × 10^6^ sperm/mL) (Table 2).

**Table 2:** Effect of *Rosmarinus officinalis *on histometric parameters and sperm Dynamics of rats

**Treatment**	**Dose**	**Total count (Total sperm of rat × 10** ^6^ **)**	**Sperm motility (%)**	**Sperm Livability (%)**
Control group	10 mL/Kg b.w	59.50 ± 0.29	63.13 ± 1.86	67.13 ± 1.66
Group A (Rosmarinus officinalis)	50 mg /Kg b.w	58.30 ± 0.33	62.14 ± 0.98	65.43 ± 0.81
Group B (Rosmarinus officinalis)	100 mg/Kg b.w	60.10 ± 0.19	59.14 ± 0.40	64.00 ± 0.30

*Rosmarinus officinalis *extract were administered orally by gavage route for 60 days. Values are mean ± SEM. Eight rats were included per group. *p < 0.05; ** p < 0.01; *** p < 0.001, significantly compared to control group, Paired t-test

*Effect on motility and viability of sperm*

In rats from control group, Vasa deferentia sperm exhibited rapid progressive motility and it was lasted for about 1 h and 20 min. In the rat treated with *Rosmarinus officinalis *at doses of 50 and 100 mg/Kg b.w, the sperms showed the same progressive motility for 75 ± 4 min. 

Although the motility and viability of sperm were declined concurrently following the dose increasing, there was no significant difference between the percentage of sperm motility and viability of both treatment groups in comparison with control group (Table 2).

*Effect on testicular cell population*

Histological findings showed that there is a significant difference between the number of Spermatogonia at both doses of 50 and 100 mg/Kg b.w (76.13 ± 3.88 and 65.25 ± 2.22, respectively), Spermatocyte at dose of 50 mg/Kg b.w (91.25 ± 2.46) and Spermatid cells at dose of 100 mg/Kg b.w (246.13 ± 3.07), compared to the control group (Table 3). However, the number of the Spermatozoid and Sertoli cells did not changed significantly in comparison with control group at the same time. Leydig cells were also increased significantly at the dose of 100 mg/Kg b.w (34.25 ± 2.15) and insignificantly at dose of 50 mg/Kg b.w (26.75 ± 2.16) in comparison with the control group (Table 3).

**Table 3:** Effect of *Rosmarinus officinalis *on testicular cell population dynamics of rats

**Treatment**	**Dose**	**Spermatogonia**	**Spermatocyte**	**Spermatid**	**Spermatozoid**	**Sertoli cell**	**Leydig cell**
Control group	10 mL/Kg b.w	48.63 ± 2.49	67.50 ± 4.22	215.00 ± 17.51	146.63 ± 8.48	11.63 ± 0.65	19.75 ± 4.90
Group A (Rosmarinus officinalis)	50 mg /Kg b.w	76.13 ± 3.88*	91.25 ± 2.46*	353.50 ± 4.65	129.50 ± 1.83	15.00 ± 2.43	26.75 ± 2.16
Group B (Rosmarinus officinalis)	100 mg/Kg b.w	65.25 ± 2.22*	75.75 ± 2.90	246.13 ± 3.07*	135.50 ± 2.40	13.88 ± 1.02	34.25 ± 2.15*

*Rosmarinus officinalis *extracts were administered orally by gavage route for 60 days. Values are mean ± SEM. Eight rats were included in each group. *p < 0.05; ** p < 0.01; *** p < 0.001, significantly compared to control group, Paired t-test

*Testes, body weight and their ratio*

The results of this study revealed that there were no significant differences found between the initial and final proportion of testes weight to body weights of rat treated with *Rosmarinus* *officinalis *at both doses compared to control group. Meanwhile, the extract of *Rosmarinus* *officinalis *at the doses of 50 and 100 mg/Kg b.w had no effect on the ratio of Testis Weights to

body weight (Table 4). 

**Table 4:** Effect of *Rosmarinus officinalis *on testis weights to body weight ratio of rats

**Treatment**	**Dose**	**Testis weight to body weight ratio**
Control group	10 mL/Kg b.w	0.5125 ± 0.0240
Group A (Rosmarinus officinalis)	50 mg /Kg b.w	0.4957 ± 0.0158
Group B (Rosmarinus officinalis)	100 mg/Kg b.w	0.5114 ± 0.0246

*Rosmarinus officinalis *extracts were administered orally by gavage route for 60 days. Values are the mean ± SEM. Ten rats were included in each group. *p < 0.05; ** p < 0.01; *** p < 0.001, significantly compared to control group, Paired t-test

The aim of the present research was to evaluate the effects of *Rosmarinus officinalis *extract on hormonal and cellular function of testes in rats. *Rosmarinus officinalis *is a typical Mediterranean species, which is now cultivated all over the world. It presents a high genetic variability, which is reflected in the chemical composition of the different individuals, and probably in its biological activity. This plant is used as a spice and traditional medicine around the world, as well as in cosmetics. In medicine, the extract of *Rosmarinus officinalis *has been attended due to its antimicrobial, anti-inflammatory and antioxidative component (8).

 The antioxidant properties of *Rosmarinus* *officinalis *have been well documented, and there are several reports that have established carnosic acid as the major phenolic diterpenoid present in rosemary leaves with antioxidant activity (9). Recently, this phenolic compound has attracted wide interest as a potential therapeutic agent against several diseases, and research was started to investigate new biological activities. Studies showed that it has chemopreventive, antineoplastic (10-11) and radioprotectiveantimutagenic effects (12).

 Hardy *et al. *and McLachlan *et al. *showed that the dramatic increase in adult Leydig cell number after neonatal PTU (6-propyl-2- thiouracil) treatment is counterbalanced by a permanent decline in Leydig cell steroidogenic function, producing no net change in peripheral testosterone levels. It seems that *Rosmarinus* *officinalis *in lower doses than 250 mg/Kg b.w reduces the testosterone. This reduction increases LH indirectly which causes the Leydig cell to increase. However, dysfunction of Leydig cells and lack of proper and adequate secretion of testosterone hormone are the main reasons for the reducing of testosterone hormone. On the other hand, FSH is also increased concurrently with the initial increase in LH. FSH increases spermatogenesis process and transforming of spermatogoni to spermatid. Although the number of these two recent cells is increased, spermatogenesis process is declined due to the lack of testosterone hormone and the population of spermatozoa cells eventually is decreased steeply (13-14).

In the present study, findings showed that there is no significant difference in the view of total number of spermatozoa between the treatment (50, 100 mg/Kg b.w) and control groups. Number of sperms had been decreased in treatment groups; however it was not significant. Nusier *et al. *investigated the effect of ingesting an extract of *Rosmarinus officinalis *leaves on fertility and sexual maturation in the male rat. Their result showed that the administration of *Rosmarinus officinalis *extract at doses of 250 and 500 mg/Kg b.w caused a significant decrease in the germinal cell population (15). Our study showed that sperm motility was not significantly decreased in treatment groups in comparison with control. Although the sperm motility was declined in both treatment groups, it was not significant. However, Nusier *et al.* (2007) showed that sperm motility at dose of 500 mg/Kg b.w in cauda epididymis, sperm density, seminiferous tubule diameter, Leydig cell nuclear diameter, and epithelial cell height in epididymides (cauda and caput) and seminal vesicles were significantly decreased in treatment groups in comparison with controls (15). In addition, our results showed that there is no significant difference between all groups in the view of sperm viability.

 Histological investigations showed that administration of *Rosmarinus officinalis *extract at both treatment groups caused a significant decrease in the germinal cell population. Spermatocytes (primary and secondary), spermatids and spermatogonia were decreased significantly. In addition, the numbers of Leydig cells were also significantly increased at 100 mg/ Kg b.w. The number of spermatozoa and sertoli cells, however, was not significantly different between the treatment and control groups. 

Findings of Nusier *et al. *showed that the administration of *Rosmarinus officinalis *extract had no significant effect on the body weights of treated males in comparison with control group. However, the absolute and relative weights of testes, epididymides, seminal vesicles, ventral prostates, and vas deferens were significantly reduced at doses of 500 mg/Kg b.w. Nevertheless, our results do not show considerable changes of sexual organ weights of male rats (15). Comparative hormonal studies of present study illustrated that there is a significant difference between the treatment and control groups. Testosterone hormone was declined significantly in both treatment groups in comparison with control group. This difference was even extremely significant at 100 mg/Kg b.w.

*Rosmarinus officinalis *has active components include Caffeic acid derivatives, Diterpenes (bitter) Flavonoids, phenolic compounds, Triterpenes and Volatile oil (16-17). Pathak *et al. *identified four new compounds from the stems of Dalbergia cochinchinensis which are 9-hydroxy-6, 7-dimethoxydalbergiquinol, 6-hydroxy-2, 7-dimethoxyneoflavene, 6, 4′-dihydroxy-7-methoxyflavan and 2, 2′, 5-trihydroxy-4-methoxybenzophenone, in addition to eight known phenolic compounds including 7-hydroxy-6-methoxyflavone. The first two compounds showed potent inhibitory activity towards 5α-dihydrotestosterone (DHT) which binds with an androgen receptor to form a DHT-receptor complex that causes androgendependent diseases (18).

In another survey, Gumbinger *et al. *showed that the antigonadotropic activity of Lithospermum and Lycopus species can be attributed to their phenolic components like rosmarinic acid which is common with *Rosmarinus officinalis*. These compounds represent precursors of biologically active products which are formed by an oxidation step. Complexity and instability of these products aggravates the elucidation of detailed structural properties. Therefore, the type of reaction involved had to be clarified. Among the oxidation products of phenolic substances, the corresponding quinones are found. It can be demonstrated that the reaction between quinones and unoxidized diphenols yields products with strong antigonadotropic activity. This type of reaction - the formation of quinhydrones – is proposed to be engaged in the formation of various products with antigonadotropic activity (19).

 Nahrstedt *et al. *identified two new cyclolignan derivatives from the mixture of substances obtained after oxidation of caffeic acid. Their structures were elucidated by spectroscopic methods as 2, 3-dicarboxy-6, 7-dihydroxy-1-(3›, 4’-dihydroxy)-phenyl-1, 2-dihydronaphthalene and 3-carboxy-6,7-dihydroxy-1-(3›, 4’-dihydroxy)-phenylnaphthalene. They exhibit antigonadotropic activity as do the extracts of crude drugs of Lycopus europaeus and Lithospermum officinale after oxidation by plant enzymes (20). Of course, since the permeability characteristics of blood testes barrier could be determinant in reproductive effects of drugs and environmental chemicals, it is recommended that each of active derivatives should be tested regarding to blood testes barrier (21). In conclusion, the results suggest that the extract of *Rosmarinus officinalis *may have antiandrogenic activity at doses of 50 and 100 mg/Kg b.w on fertility in male rats indicating the possibility of developing herbal male contraceptive. However, these doses of *Rosmarinus officinalis *cannot affect the spermatogenesis process. Further studies are needed to investigate the effect of *Rosmarinus officinalis *on fertility in male rats at more several doses and to determine its mechanism of action.
